# Supplementary material for: High-performance protocol for ultra-short DNA sequencing using Oxford Nanopore Technology (ONT)
Source: PLoS One. 2025 Apr 29;20(4):e0318040. doi: 10.1371/journal.pone.0318040 (PMC12040124; doi:10.1371/journal.pone.0318040)
Supplement: S1 File — dx.doi.org/10.17504/protocols.io.dm6gp973jvzp/v1. (PDF) [file pone.0318040.s001.pdf]

Feb 26, 2025

High-Performance Protocol for ONT Library Preparation

DOI
dx.doi.org/10.17504/protocols.io.dm6gp973jvzp/v1

Lukas Žemaitis[1], Rūta Palepšienė[1], Simonas Juzėnas[1], Gediminas Alzbutas[1,2], Pierre-Yves Burgi[3], Thomas Heinis[4], Jérôme Charmet[5], Silvia Angeloni Suter[5], Martin Jost[6], Renaldas Raišutis[7], Friedrich Simmel[8], Ignas Galminas[1,9]

[1]Department of DNA data storage, Genomika, Kaunas, Lithuania;
[2]Institute for Digestive Research, Academy of Medicine, Lithuanian University of Health Sciences, Kaunas, Lithuania;
[3]Department of Information and Communication Systems and Technologies, University of Geneva, Geneva, Switzerland;
[4]Department of Computing, Imperial College London, London, United Kingdom;
[5]School of Engineering HE-Arc Ingénierie, HES-SO University of Applied Sciences Western Switzerland, Neuchâtel, Switzerland;
[6]MABEAL GmbH, Graz, Austria; [7]Ultrasound Research Institute, Kaunas University of Technology, Kaunas, Lithuania;
[8]Department of Bioscience, TU Munich, School of Natural Sciences, Garching, Germany;
[9]Faculty of Natural Sciences, Vytautas Magnus University, Kaunas, Lithuania.

R Rūta Palepšienė
Department of DNA data storage, UAB Genomika

OPEN ACCESS

DOI: dx.doi.org/10.17504/protocols.io.dm6gp973jvzp/v1

Protocol Citation: Lukas Žemaitis, Rūta Palepšienė, Simonas Juzėnas, Gediminas Alzbutas, Pierre-Yves Burgi, Thomas Heinis, Jérôme Charmet, Silvia Angeloni Suter, Martin Jost, Renaldas Raišutis, Friedrich Simmel, Ignas Galminas 2025. High-Performance Protocol for ONT Library Preparation. **protocols.io** https://dx.doi.org/10.17504/protocols.io.dm6gp973jvzp/v1

License: This is an open access protocol distributed under the terms of the **Creative Commons Attribution License**, which permits unrestricted use, distribution, and reproduction in any medium, provided the original author and source are credited

Protocol status: Working
We use this protocol and it's working

Created: February 25, 2025

Last Modified: February 26, 2025

Protocol Integer ID: 123413

Keywords: DNA end preparation, Adapter ligation, Purification and elution, Flongle Flow Cell Loading and Sequencing, Flow Cell Priming, Library Preparation for Sequencing

Funders Acknowledgements:
European Innovation Council Pathfinder Program
Grant ID: 101115389

Abstract

This protocol presents a refined version of the Oxford Nanopore Technologies (ONT) Ligation Sequencing amplicons V14 (SQK-LSK114) protocol, optimized for the sequencing of short and ultra-short DNA fragments. It maintains the use of reagents required for standard ONT library preparation while enhancing DNA yield and improving sequencing performance.

The protocol can be successfully used for all types of Flow Cells, but the Flow Cell priming and loaded library volumes must be adjusted accordingly.

Materials

- 0.2 mL PCR tube
- 1.5 mL RNase- and DNase-free low-bind tube
- Milli-Q water (Thermo Fisher Scientific)
- Ultra II End-Prep Reaction Buffer (E7546S, NEB)
- Ultra II End-Prep Enzyme Mix (E7546S, NEB)
- Ligation Buffer (LNB, ONT)
- Quick T4 DNA Ligase (E6056S, NEB)
- Ligation Adapters (LA, ONT)
- Small Fragment Buffer (SFB, ONT)
- Elution Buffer (EB, ONT)
- Qubit HS dsDNA kit (Thermo Fisher Scientific)
- Flongle (ONT) flow cell
- MinION Mk1B device (ONT)
- Flow Cell Flush (FCF, ONT)
- Flow Cell Tether (FCT, ONT)
- Sequencing Buffer (SB, ONT)
- Library Beads (LIB, ONT)

NEBNext Ultra II End Repair/dA-Tailing Module - 24 rxns **New England Biolabs** **Catalog #**E7546S

NEBNext Quick Ligation Module **New England Biolabs** **Catalog #**E6056S

Before start

- Thaw all the reagents and keep them on ice throughout the protocol.
- Prior to use, thoroughly mix all components by vortexing, except for the End-Prep Enzyme Mix and Quick T4 DNA Ligase.
- Perform all centrifugation steps at room temperature.

Note

Note: "ONT" refers to reagents from the standard SQK-LSK114 kit provided by Oxford Nanopore Technologies.

DNA End Preparation

10m

1 Transfer [M] 250 Mass Percent of the dsDNA to a clean 0.2 mL PCR tube, adjusting the volume based on DNA concentration.

2 Add the following components sequentially, mixing by pipetting approximately 10 times after each addition:

A	B
**Component**	**Volume**
dsDNA	X µL (250 fmol)
Milli-Q water (Thermo Fisher Scientific)	X µL (to 50 µL)
Ultra II End-Prep Reaction Buffer (E7546S, NEB)	7 µL
Ultra II End-Prep Enzyme Mix (E7546S, NEB)	3 µL
**Total volume:**	**60 µL**

3 Briefly centrifuge the tube and place it into a thermal cycler with a heated lid.

4 Run the following thermal cycling program:
20 °C for 00:05:00 .
65 °C for 00:05:00 .

10m

Adapter Ligation

20m

5 After incubation, transfer the reaction mixture to a 1.5 mL RNase- and DNase-free low-bind tube.

6 Add the following components in the specified order, mixing by pipetting approximately 10 times after each addition:

A	B
**Component**	**Volume**
End-prepped DNA	60 µL
Ligation Buffer (LNB, ONT)	25 µL
Quick T4 DNA Ligase (E6056S, NEB)	10 µL
Ligation Adapters (LA, ONT)	5 µL

A	B
**Total volume**	**100 µL**

7 Incubate the reaction at Room temperature for 00:20:00 . 20m

Purification and Elution

23m

8 Add AMPure XP Beads (AXP, ONT) to the reaction tube based on the DNA length:

- 90 µL for fragments < 100 bp.
- 50 µL for fragments > 100 bp.

9 Mix by inverting the tube for 00:05:00 . 5m

10 Briefly centrifuge the tube, then place it on a magnetic rack for 00:03:00 to pellet the beads. 3m

11 While keeping the tube on the magnet, carefully remove the supernatant.

12 Add 250 µL of Small Fragment Buffer (SFB, ONT) to the tube.

13 Remove the tube from the magnet and gently rotate for at least 00:03:00 , occasionally flicking to ensure even bead distribution. 3m

14 Briefly centrifuge the tube and place it back on the magnet for 00:03:00 , then remove the supernatant. 3m

15 Repeat the wash step with 250 µL of SFB following the same procedure.

16 After the final wash, briefly centrifuge the tube, then place it on the magnet to remove any remaining supernatant.

17 Allow the bead pellet to dry for 00:01:00 before removing the tube from the magnet. 1m

18 Add 12 µL of Elution Buffer (EB, ONT) to the tube and mix thoroughly by pipetting.

19 Incubate at Room temperature for 00:05:00 . 5m

20 Place the tube back on the magnet for 00:03:00 to pellet the beads. 3m

21 Transfer the supernatant containing the prepared library to a new 1.5 mL RNase- and DNase-free tube and place On ice .

22 Measure DNA concentration using the Qubit HS dsDNA kit (Thermo Fisher Scientific).

Flongle Flow Cell Loading and Sequencing: Flow Cell Preparation

23 Insert a Flongle (ONT) flow cell into a MinION Mk1B device (ONT).

24 Perform a quality check using MinKNOW software.

25 Select only flow cells with ≥ 50 active pores for sequencing.

Flongle Flow Cell Loading and Sequencing: Flow Cell Priming 5m

26 Prepare the flow cell priming mix by combining the following components in a 1.5 mL tube, then immediately place On ice :

A	B
**Component**	**Volume**
Flow Cell Flush (FCF, ONT)	117 µL
Flow Cell Tether (FCT, ONT)	3 µL
**Total volume:**	**120 µL**

27 Peel back the seal tab of the Flongle flow cell to expose the sample port.

28 Secure the seal tab by adhering it to the MinION lid using its adhesive strip.

29 Carefully load the priming mix into the flow cell, ensuring no air bubbles are introduced.

30 Incubate the flow cell for 00:05:00 .

5m

Flongle Flow Cell Loading and Sequencing: Library Preparation for Sequencing

10m

31 During the incubation period, prepare the sequencing mix by combining the following components:

A	B
**Component**	**Volume**
Sequencing Buffer (SB, ONT)	15 µL
Library Beads (LIB, ONT)	10 µL
Prepared Library	X µL (50 fmol)
Elution Buffer (EB, ONT)	X µL
**Total volume:**	**35 µL**

32 Incubate at Room temperature for 00:10:00 before initiating sequencing run.

10m
