# Supplementary figures and images for: High-performance protocol for ultra-short DNA sequencing using Oxford Nanopore Technology (ONT)
Source: PLoS One. 2025 Apr 29;20(4):e0318040. doi: 10.1371/journal.pone.0318040 (PMC12040124; doi:10.1371/journal.pone.0318040)

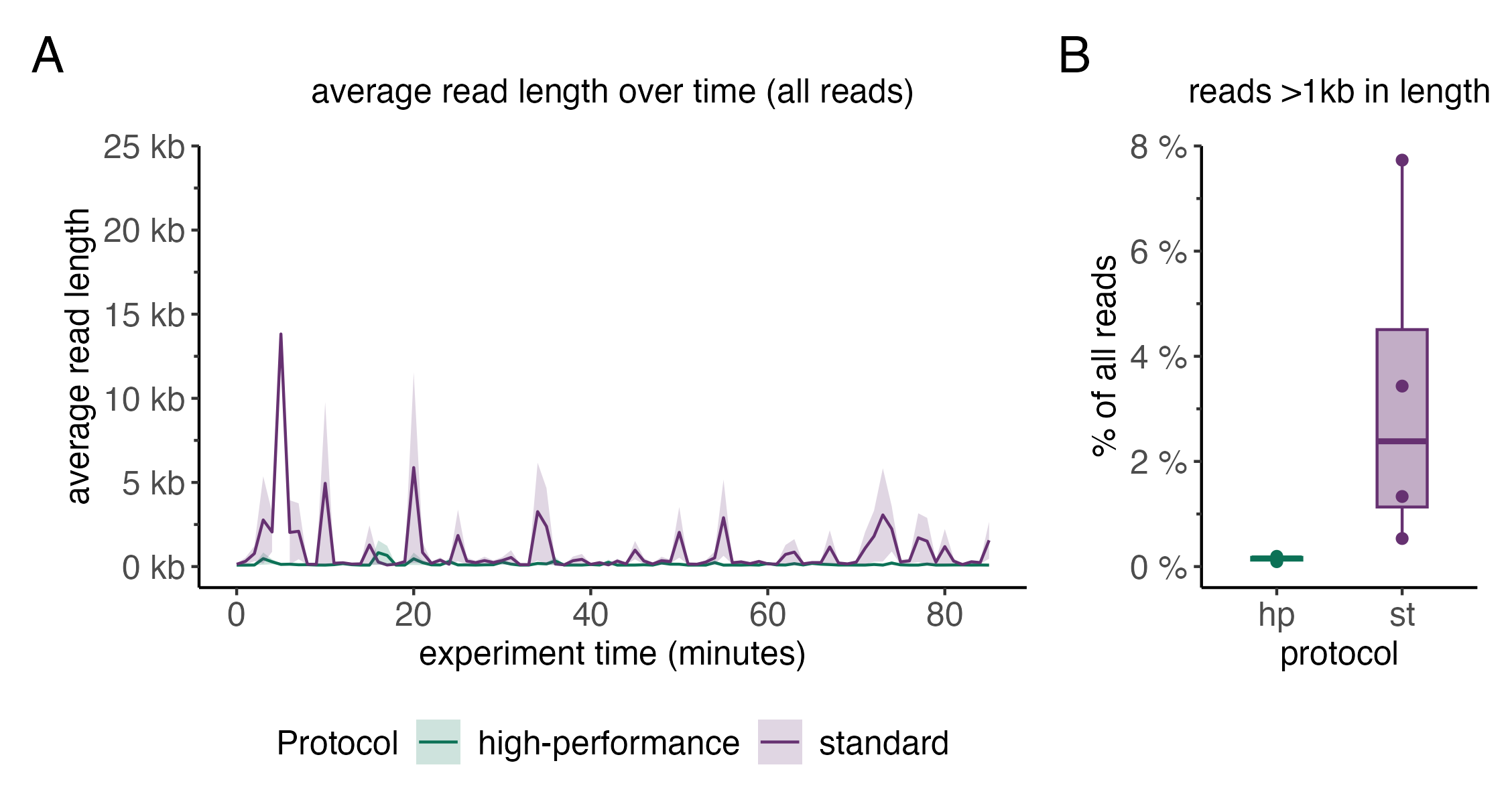

Supplement: S1 Fig — A) Average read length of sequences produced over time are more homogenous for the libraries prepared by High-performance protocol. The line graph shows the average read length with standard error (SE) for libraries prepared using the standard and High-performance protocols. B) As shown in the boxplots, libraries prepared using the standard (st) library preparation protocol have a higher fraction of reads with a length of 1 kb or more compared to those prepared using the High-performance (hp) protocol. Of note, sequencing artifacts in this case refer to unexpectedly long reads exhibiting low sequence diversity. The results represent three (N = 3) and four (N = 4) independent sequencing experiments by using the High-performance and standard ONT protocol, respectively. (TIF) [file pone.0318040.s002.tif]
